# Investigation on current situation of Chinese patent medicines for children in China based on the national drug coding database

**DOI:** 10.3389/fphar.2025.1626274

**Published:** 2025-06-12

**Authors:** Hui Liu, Ping Rong, Yatong Zhang, Yuqiong You, Qianfang Fu, Lili Cai, Juan Wei, Rong Ma

**Affiliations:** ^1^ First Teaching Hospital of Tianjin University of Traditional Chinese Medicine, Tianjin, China; ^2^ National Clinical Research Center for Chinese Medicine Acupuncture and Moxibustion, Tianjin, China

**Keywords:** national drug coding database, Chinese patent medicines, precision medicine for children, department of pediatric, traditional Chinese medicine (TCM)

## Abstract

**Introduction:**

Chinese patent medicine is a crucial component of traditional Chinese medicine, significantly promoting public health. Despite the extensive research on Chinese patent medicine for children, various issues persist in its application. Leveraging the national drug coding standard code database of the National Medical Products Administration (NMPA), this investigation delved into the accessibility of Chinese patent medicines tailored for children, offering a comprehensive overview of the current landscape in China. This analysis serves as a valuable resource for formulating targeted policies to promote the use of Chinese patent medicines for children, guiding drug selection, and facilitating the development of pediatric pharmaceuticals.

**Methods:**

Taking Chinese patent medicines with “National Medicine Approval Number Z” and “National Medicine Approval Number B” from the NMPA National Drug Coding Database as the research subjects, this study systematically analyzed the distribution, characteristics, and existing issues of Chinese patent medicines for children using descriptive statistical methods.

**Results and discussion:**

As of May 2023, 8,903 approved “National Medicine Approval Number Z” Chinese patent medicines, 951 approved “National Medicine Approval Number B” Chinese patent medicines in China, with 1,164 Chinese patent medicines for children identified. Chinese patent medicines for children are predominantly administered orally (92.87%), while external preparations are limited. The taste profile is primarily bitter or sweet, with some medications having other undesirable flavors. The therapeutic focus is skewed toward pulmonary system diseases (31.9%) and spleen-stomach diseases (26.5%). Notably, 49.66% of the medications mention Western medical disease names, and 63.92% reference traditional Chinese medicine (TCM) syndrome types. Critical gaps include unclear age-specific dosage instructions (56.70%), lack of specified treatment duration (92.01%), and incomplete safety information, with adverse reactions mainly involving the gastrointestinal system. Current issues include the limited availability of Chinese patent medicines for children, poor suitability for children, imbalanced research and supply (over-concentration in pulmonary and spleen-stomach disorders), and inadequate safety labeling in drug instructions, posing potential risks. Recommendations include developing new Chinese patent medicines for children, improving drug suitability, conducting post-marketing evaluations, and refining drug labeling to ensure safe and rational pediatric medication use.

## 1 Introduction

Chinese patent medicine is a standardized preparation formulated based on specific prescriptions and preparation processes rooted in traditional Chinese medicine theory. These formulations are approved by the National Drug Supervision and Administration Department to address disease prevention and treatment requirements. Chinese patent medicine constitutes a significant component of traditional Chinese medicine, contributing substantially to the maintenance of public health. Due to its stable nature, notable efficacy, and minimal side effects, Chinese patent medicine finds extensive application in pediatric clinical settings. Medical institutions at all levels demonstrate a higher utilization rate of Chinese patent medicines compared to Western drugs in pediatric treatments (Cheng and Li, 2016). However, emerging research on pediatric Chinese patent medicines reveals persistent challenges including limited dedicated pediatric formulations, insufficient age-appropriate dosage forms, inadequate medication information labeling, and prevalent off-label drug use (Ruan et al., 2023; Cai et al., 2022; Ren, 2023; Zhang et al., 2022). In response, multiple national departments have implemented policy initiatives to encourage R&D innovation, ensure production supply, and strengthen regulatory oversight. Technical support has been provided for pediatric drug development through real-world studies, model construction, and pharmaceutical development standardization.

This study conducts an in-depth exploration of pediatric Chinese patent medicine accessibility using the National Drug Code Master Database, systematically analyzing their current status in China. The findings aim to provide data support for targeted national incentive policies, inform drug selection processes, and guide pediatric medication research and development, ultimately ensuring safer pharmaceutical care for children.

## 2 Materials and methods

### 2.1 Data sources

Chinese patent medicines are registered in the National Drug Code Master Database of the National Medical Products Administration with approval numbers prefixed (National Medical Products Administration, 2002) ‘National Medicine Approval Number Z′ and ‘National Medicine Approval Number B' (as of May 2023).

### 2.2 Scope definition

The study defined children as natural persons under the age of 18. Chinese patent medicine for children is defined as a drug with children’s information, indications for children, or dosage in the drug name and drug package insert. Based on the requirements from the ‘Notice on Further Strengthening Medication Safety Management and Improving Rational Drug Use Practice' (National Health Commission, 2022) Regarding pediatric medication specifications (medications with explicit pediatric indications and dosage instructions in drug package inserts), pediatric Chinese patent medicines are classified into three categories based on pediatric medication information: 1) Pediatric-specific Medications: Those with functions/indications or usage/dosage exclusively for children, or containing terms like “Xiaoer” (pediatric) or “Ertong” (children) in their names; 2) Medications for Both Children and Adults: Those containing both adult and pediatric dosage specifications, or including pediatric information in their functions/indications; 3) Medications with Dosage Reduction for Children: Those with adult dosage specifications but lacking pediatric dosage information, labeled with “appropriate reduction for children” or “pediatric dosage as prescribed” in usage instructions, and/or containing pediatric information in functions/indications.

### 2.3 Search for pharmaceutical products

By the “Notice on the Uniform Replacement and Standardization of Drug Approval Number Formats” (National Medical Products Administration, 2002) Searches were conducted using the keywords ' National Medicine Approval Number Z′ and ' National Medicine Approval Number B′ in the National Drug Code Master Database (https://www.nmpa.gov.cn/datasearch/home-index.html#category=yp).

### 2.4 Drug package insert search

After merging and deduplicating the “Product Name” entries from the search results of ' National Medicine Approval Number r Z′ and ' National Medicine Approval Number B′ products, active pharmaceutical ingredients were excluded based on the ‘Details-Product Category’ in the database. Using standardized drug names as search terms, the latest drug package inserts were collected through primary channels including the China Pediatric Drug Database (https://www.dayi.org.cn/), Yaozhiwang (https://db.yaozh.com/), and Chinese Pediatric Drug Database (http://cpd.pharmadl.com/), supplemented by secondary channels such as Quick Doctor Inquiry, 39 Drug Encyclopedia, and Medical Alliance Media.

### 2.5 Establishment of Pediatric Chinese patent medicine database

Using search terms such as “child”, “year-old”, “infant”, and “baby” in the fields of “Pharmaceutical Product Name”, “Functions and Indications”, and “Usage and Dosage”, we screened drug package inserts for pediatric Chinese patent medicines. The extracted contents from included drug package inserts were recorded into Excel spreadsheets to establish the Pediatric Chinese Patent Medicine Database. The extracted information included: product name, composition, properties, functions and indications, specifications, usage and dosage, adverse reactions, usage precautions, clinical trials, and pharmacological effects.

### 2.6 Data analysis

Descriptive statistical methods were employed to analyze the distribution patterns of pediatric Chinese patent medicines, including pediatric-specific medications, medications for both children and adults and medications with dosage reduction for children. The analysis covered aspects such as route of administration, dosage forms, palatability, functions and indications, usage and dosage, and safety information. Proportional distributions for each category were calculated and presented.

Classification of Ethnic Medicines Based on the *Catalog of Chinese Ethnic Patent Medicines* compiled by the research group from Minzu University of China in 2020 (source: Compilation of Chinese Ethnomedicine Patent Formulas, 2020) Ethnic medicines were classified.

References including *Pediatrics of Traditional Chinese Medicine*, *Internal Medicine of Traditional Chinese Medicine*, *Orthopedics and Traumatology of Traditional Chinese Medicine*, *Surgery of Traditional Chinese Medicine*, *Otorhinolaryngology of Traditional Chinese Medicine*, *Ophthalmology of Traditional Chinese Medicine*, and the *Dictionary of Clinical Drugs in China · Volume of Chinese Patent Formulations* were used to classify the functions and indications of pediatric Chinese patent medicines. For medicines with multiple clinical therapeutic applications, their primary therapeutic purpose was selected for classification.

Adverse reaction manifestations in drug package inserts were summarized and statistically analyzed according to the WHO Adverse Reaction Terminology Set: System-Organ Class Code Retrieval Directory.

The names of included pediatric Chinese patent medicines were cross-referenced against the 2020 edition of the Pharmacopoeia of the People’s Republic of China, the 2018 edition of the National Essential Medicines Directory of China, and the 2024 edition of the National Basic Medical Insurance, Work Injury Insurance, and Maternity Insurance Drug Directory (collectively referred to as the ‘Three Major Directories').

## 3 Results

### 3.1 Quantity of pediatric Chinese patent medicines

As of May 2023, China’s drug regulatory system contained 57,826 domestic drug approval numbers under ‘National Medicine Approval Number Z′ and 83 imported drug approval numbers, along with 990‘National Medicine Approval Number B'. After consolidating and deduplicating product names, 9,847 Chinese patent medicines (excluding raw drug materials) have been approved for market in China. Among these, 1,164 (11.82%) are pediatric Chinese patent medicines. Out of the 1,164 pediatric Chinese patent medicines, 1,075 are Chinese patent medicines (National Medicine Approval Number Z) for children, with an additional 90 Chinese patent medicines (National Medicine Approval Number B) for children due to Yanggan Mingmu Tablets holding two distinct approval numbers (Z20123058 and B20020343). This includes 524 pediatric-specific medications (5.32%), 213 medications for both children and adults (2.16%), and 427 medications with dosage reduction for children (4.34%).

### 3.2 Basic information on pediatric Chinese patent medicines

Analysis of 1,164 pediatric Chinese patent medicines revealed 1,153 domestic pharmaceutical products (98.97%) and 12 imported pharmaceutical products (1.03%). Ethnic medicines accounted for 40 types (3.44%), primarily including Tibetan, Yi, and Miao ethnic medicines. For detailed distributions, see Table 1. The market comprises 690 exclusive pharmaceutical products (59.28%), 142 products (12.20%) manufactured by two enterprises, and 332 products (28.52%) produced by three or more manufacturers. See Table 2 for details. Exclusive products dominate China’s pediatric Chinese patent medicine market. The analysis revealed 747 prescription drug varieties (64.18%), 283 over-the-counter varieties (24.31%), and 134 dual-status medications (11.51%), as detailed in Table 3. Pediatric Chinese patent medicines in China are primarily classified as prescription drugs. Over the past decade (2013–2022), only eight new pediatric-specific medications among Chinese patent medicines have been approved for market release in China, with five varieties specifically indicated for treating pediatric neurological disorders.

**TABLE 1:** Imported herbal medicines and ethnic herbal medicines analysis of Chinese patent medicines for children.

Category	Pharmaceutical Product Name
Imported Medicines	Bao Ying Dan, Myrtol Standardized Enteric-coated Capsules (Pediatric), Du Tong Xie Wan, Fei Er Wan He Wei Zheng Chang Wan, Kyoto Ninjiom Pei Pa Koa, Kang Fu Zhi Xie Pian, Sinupret Oral Drops, Po Chai Pills, Wu Ta Biao Xing Jun San, Xiao Er Zhu Po San (formerly known as Zhu Po Hou Zao San), Seirogan
Tibetan Ethnomedicine	An’erning Granules, Feire Puqing Powder, Liuwei Nengxiao Capsules, Qiwei Suantengguo Pills, Sanchen Capsules, Sanchen Powder, Shanhu Qishiwei Pills, Xiao’er Shuangqing Granules, Yigan Huoxue Mingmu Pills
Yi Ethnomedicine	Changshu Zhixie Capsules, Changweishu Capsules, Chuanluotong Capsules, Henggu Gushang Yuheji (Ancient Bone Trauma Healer), Jinshile Eye Drops, Longze Xiongdan Capsules (Bear Bile Pills), Sechang Zhixie Powder, Shijiaocao Kechuan Granules, Zhen Xiongdan Pills
Miao Ethnic Medicine	Erpixing Granules, Feilike Mixture, Kaihoujian Spray (Child-specific), Xiaoer Gonglao Antidiarrheal Granules, Xingpi Capsule, Xingpi Yang’er Granules, Zhuxiao Ointment, Yifei Zhike Capsule
Mongolian Ethnic Medicine	Bianlei Granules, Jiegeng Bawei Granules, Sanchen Pill, Xiaoer Qingfei Bawei Pill, Xiaoer Shikou Powder
Dai Ethnic Medicine	Shanzha Neijin Capsule, Shanzha Neijin Oral Liquids, Yinqin Capsule, Shenbei Zhike Granules
Korean ethnic medicine	Weixuening Mixture, Pediatric Cough Syrup
She ethnic medicine	Pediatric Seven-Star Tea Syrup
Tujia ethnic medicine	Pediatric Cold Granules
Uyghur ethnic medicine	Six-Ingredient Saffron Oral Liquid

**TABLE 2:** Pharmaceutical manufacturer analysis of Chinese patent medicines for children.

Number of manufacturing enterprises	Pediatric-specific medications	Medications for both children and adults	Medications with dosage reduction for children	Proportion/%
1	297	155	238	59.28
2	69	21	52	12.20
3	30	11	19	5.15
>3	128	26	118	23.37

**TABLE 3:** Prescription drug and Over-the-Counter drug analysis of Chinese patent medicines for children.

Category	Pediatric-specific medications	Medications for both children and adults	Medications with dosage reduction for children	Proportion/%
Prescription drugs	322	131	294	64.18
Dual-status	45	25	64	11.51
Over-the-counter drugs	157	57	69	24.31

### 3.3 Routes of administration and dosage forms

Analysis of 1,164 pediatric Chinese patent medicines revealed the following distribution of administration routes and dosage forms: oral administration (1,081, 92.87%) primarily including granules, pills, and tablet formulations; topical medications (27, 2.32%) comprising plaster preparations, ointments, and lozenges; combined oral/topical medications (24, 2.06%) including pills, tablets, and powder formulations; injectable preparations (14, 1.20%), rectal administration (6, 0.52%), oral mucosal medications (5, 0.43%), ophthalmic preparations (4, 0.34%), and inhalation medications (3, 0.26%) (see Table 4 for details). Chinese patent medicines (National Medicine Approval Number B) for children was mainly administered orally (81/90, 90.0%), and the dosage forms were mainly oral liquid (36/90, 40.0%) and granule (21/90, 23.3%). Pediatric Chinese patent medicines predominantly employ oral administration, with principal dosage forms consisting of granules, pills, tablets, capsule preparations, and oral liquids, while topical, rectal, and inhalation administration methods remain underutilized.

**TABLE 4:** Administration and dosage form analysis of Chinese patent medicines for children.

Route of administration	Dosage forms	Pediatric-specific medications	Medications for both children and adults	Medications with dosage reduction for children	Proportion/%
Oral	Granules	118	38	60	18.56
	Pills	86	37	66	16.24
	Tablet Formulation	53	32	94	15.38
	Capsule Preparation	15	28	77	10.31
	Oral Liquids	70	14	35	10.22
	Powder Formulation	83	9	16	9.28
	Syrup	36	10	22	5.84
	Mixture	15	7	8	2.58
	Electuary	6	0	5	0.95
	Medicinal Tea	4	4	1	0.77
	Chewable Tablets	4	1	3	0.69
	Soft Capsule Preparation	0	1	4	0.43
	Troche	3	1	0	0.34
	Effervescent Tablets	2	1	0	0.26
	Dropping Pills	1	0	1	0.17
	Tinctures	0	0	2	0.17
	Dispersible Tablets	0	0	2	0.17
	Medicinal Ferments	0	0	2	0.17
	Bagged Tea	1	0	0	0.09
	Gelatins	0	0	1	0.09
	Oral Gels	1	0	0	0.09
	Distillates	0	0	1	0.09
Oral/Topical Use	Pills	0	6	2	0.69
	Tablet Formulation	0	3	2	0.43
	Powder Formulation	2	2	1	0.43
	Capsule Preparation	1	1	1	0.26
	Liniments	0	0	1	0.09
	Tinctures	0	0	1	0.09
	Troche	0	1	0	0.09
Buccal Medications	Sprays	1	0	1	0.17
	Oral Gums	0	1	0	0.09
	Aerosols	0	0	1	0.09
	Powder Formulation	0	0	1	0.09
Topical Use	Adhesive Plasters	6	2	0	0.69
	Ointments	3	1	0	0.34
	Troche	0	0	2	0.17
	Medicinal Plasters	2	0	0	0.17
	Powder Formulation	1	1	0	0.17
	Adhesive	2	0	0	0.17
	Liniments	1	0	0	0.09
	Tinctures	0	1	0	0.09
	Capsule Preparation	0	0	1	0.09
	Gel	0	0	1	0.09
	External film	1	0	0	0.09
	Pills	0	0	1	0.09
	Lotion	1	0	0	0.09
Inhalation	Aerosols	0	1	1	0.17
	Sprays	0	1	0	0.09
Ophthalmic	Eye drops	0	4	0	0.34
Rectal	Suppository	4	1	1	0.52
Injection	Injectable	1	4	9	1.20

### 3.4 Palatability

Based on formulation characteristics, we analyzed the palatability of 1,112 pediatric oral Chinese patent medicines (Table 5). The taste of 82 kinds of oral Chinese patent medicines (National Medicine Approval Number B) for children was mainly sweet (66/82, 80.5%) and bitter (42/82, 51.2%). The predominant tastes of 1,112 pediatric oral Chinese patent medicines were bitter (66.73%) and sweet (53.96%), while unpleasant flavors including sour, salty, spicy, cool, astringent, numbing, fishy, and metallic odors were also observed, potentially impacting medication adherence in children.

**TABLE 5:** Taste analysis of oral Chinese patent medicines for children.

Palatability		Pediatric-specific medications	Medications for both children and adults	Medications with dosage reduction for children	Proportion/%
Bitter	Slightly bitter	216	60	156	38.85
	Bitter	112	63	135	27.88
Sweet	Sweet	282	82	152	46.40
	Slightly sweet	42	16	26	7.55
Sour	Slightly sour	28	13	13	4.86
	Sour	31	9	13	4.77
Salty	Salty	7	6	10	2.07
	Slightly salty	5	2	5	1.08
Astringent	Astringent	19	16	38	6.56
	Slightly astringent	18	6	14	3.42
Pungent	Acrid	42	16	29	7.82
	Slightly acrid	16	12	14	3.78
	Pungent	1	5	6	1.08
	Spicy	2	2	6	0.90
	Slightly spicy	1	0	0	0.09
	Slightly pungent	0	1	0	0.09
Cool	Cool	39	14	42	8.54
	Slightly cool	5	4	2	0.99
Numbness	Numbness	3	11	8	1.98
	Slight numbness	4	2	2	0.72
Fishy	Slightly fishy	10	5	6	1.89
	Fishy	2	2	4	0.72
	Metallic odor	1	0	0	0.09

### 3.5 Functions and indications

A summary was conducted on the functions and indications of 1,164 types of pediatric Chinese patent medicines. Regarding Western medical disease name mentions: 578 medications (49.66%) explicitly mentioned Western disease names, including 211 pediatric-specific medications, 119 medications for both children and adults, and 248 medications with dosage reduction for children. 586 medications (50.34%) did not explicitly mention Western disease names, including 313 pediatric-specific medications, 94 medications for both children and adults and 179 medications with dosage reduction for children.

For TCM syndrome pattern mentions: 744 medications (63.92%) explicitly mentioned TCM syndromes, including 330 pediatric-specific medications, 152 medications for both children and adults and 262 medications with dosage reduction for children. 420 medications (36.08%) did not explicitly mention TCM syndromes, including 194 pediatric-specific medications, 61 medications for both children and adults and 165 medications with dosage reduction for children.

The distribution of treated disease categories (Table 6) reveals that pediatric Chinese patent medicines primarily address pulmonary disorders (343, 31.9%) and splenic disorders (285, 26.5%), followed by cardio-cerebral disorders (134, 12.5%). Chinese patent medicines (National Medicine Approval Number B) for children primarily address splenic disorders (34/90, 37.8%) and cardio-cerebral disorders (13/90, 14.4%). The scope also extends to disorders of qi, blood, and body fluids, ear-nose-throat disorders, hepatobiliary disorders, surgical diseases, renal disorders, orthopedic and traumatic diseases, infectious diseases, and parasitic diseases. Notably, medications for both children and adults and those requiring dosage reduction for children constitute the majority of therapeutic interventions.

**TABLE 6:** Diseases analysis of Chinese patent medicines for children.

System	Pediatric-specific medications	Medications for both children and adults	Medications with dosage reduction for children	Proportion/%
Pulmonary Disorders	174	54	125	30.33
Splenic Disorders	204	45	72	27.58
cardio-cerebral disorders	76	28	43	12.63
Disorders of Qi, Blood, and Body Fluids	9	25	33	5.76
Ear, Nose, and Throat Disorders	18	12	29	5.07
Hepatobiliary Disorders	3	5	41	4.21
Surgical Diseases	11	10	24	3.87
Others	8	4	14	2.23
Renal Disorders	4	1	17	1.89
Orthopedic and Traumatic Diseases	0	14	6	1.72
Infectious Diseases	8	0	9	1.46
Ophthalmic Disorders	1	11	2	1.20
Parasitic Diseases	8	1	4	1.12
Musculoskeletal and Meridian Disorders	0	3	8	0.95

### 3.6 Usage and dosage

An analysis of usage and dosage for 1,164 pediatric Chinese patent medicines revealed: three formulations (0.26%) calculated dosage by children’s weight, including Tanreqing Injection (0.3–0.5 mL/kg), Yinqin Capsule (1 capsule per 10 kg), and Xiyanping Injection (5–10 mg/kg); 660 formulations (56.70%) lacked explicit age-based dosage stratification, comprising 156 pediatric-specific medications, 77 medications for both children and adults and 427 medications with dosage reduction for children; 501 formulations (43.04%) provided explicit age-stratified dosage instructions, including 368 pediatric-specific medications and 133 medications for both children and adults. Among Chinese patent medicines (National Medicine Approval Number B) for children, 56 formulations (62.2%) lacked explicit age-based dosage stratification, 34 formulations (37.8%) provided explicit age-stratified dosage instructions.

Analysis of age stratification patterns among 501 pharmaceutical products with explicit age-differentiated dosage specifications (Table 7) revealed inconsistent age categorization for pediatric use. Age brackets with frequency 40 included: <1 year, 1–3 years, 1–2 years, >3 years, 3–5 years, >5 years, <3 years, 1 year, and 4–6 years.

**TABLE 7:** Age group analysis on the usage and dosage of Chinese patent medicines for children.

Age group	Pediatric-specific medications (types)	Medications for both children and adults (types)	Proportion/%
Under 1 year	135	19	30.74
1–3 years	94	22	23.15
1–2 years	53	7	11.98
Over 3 years	37	8	8.98
3–5 years	36	7	8.58
Over 5 years	32	9	8.18
Under 3 years	21	20	8.18
1 year	32	9	8.18
4–6 years	34	6	7.98
3–7 years	19	16	6.99
Over 10 years	27	6	6.59
Over 7 years	20	12	6.39

Note: Age groups with occurrence frequencies <30 are excluded from the table.

Notably, 23 pediatric Chinese patent medicines (1.98%) employed non-standard age descriptors such as “infant,” “neonate,” “newborn,” or “young child” to denote specific age ranges. Among these, 14 were Pediatric-specific Medications and nine were Medications for Both Children and Adults. For instance, a diarrhea-treating granule specifies: “Adults: 10g/dose, young children: 5g/dose, infants: 3g/dose.” Furthermore, 69 pediatric Chinese patent medicines (5.93%) exhibited incomplete age stratification – 8 Pediatric-specific Medications and 61 Medications for Both Children and Adults. A representative example is an antitussive syrup for wind-heat cough with dosage guidelines: “one to two years: 5mL/dose, 3–5 years: 10mL/dose, adults: 20–25 mL/dose,” omitting dosage information for children aged 5–17 years.

Treatment course specifications: 1,071 pharmaceutical products (92.01%) lacked treatment course labeling, including 480 pediatric-specific medications, 192 medications for both children and adults and 399 medications with dosage reduction for children. 93 pharmaceutical products (7.99%) specified treatment courses, including 44 pediatric-specific medications, 21 medications for both children and adults and 28 medications with dosage reduction for children. Among Chinese patent medicines (National Medicine Approval Number B) for children, 81 pharmaceutical products (90.00%) lacked treatment course labeling. Treatment courses were predominantly within 7 days, primarily used for respiratory system diseases (common cold, cough) and gastrointestinal disorders (diarrhea).

### 3.7 Safety information

Statistical analysis of safety information from 1,164 pediatric Chinese patent medicines (Table 8) revealed: 161 pharmaceutical products (13.83%) had documented adverse reactions, while 1,003 products (86.17%) showed unspecified or missing adverse reaction information; 338 products (29.04%) listed contraindications, compared to 826 products (70.96%) with unspecified or missing contraindication data; 891 products (76.55%) provided usage precautions, while 273 products (23.45%) lacked such specifications; 570 products (48.97%) included drug interaction guidelines, whereas 594 products (51.03%) omitted drug interaction information. Among Chinese patent medicines (National Medicine Approval Number B) for children, 78 products (86.7%) showed unspecified or missing adverse reaction information, 51 products (56.7%) with unspecified or missing contraindication data, six products (6.73%) lacked usage precautions, 39 products (43.3%) omitted drug interaction information.

**TABLE 8:** Safety information labeling of Chinese patent medicines for children.

Category		Pediatric-specific medications	Medications for both children and adults	Medications with dosage reduction for children	Proportion/%
Adverse reactions	Labeled	63	50	48	13.83
	Missing	3	4	2	0.77
	Not yet specified	458	159	377	85.40
Contraindications	Labeled	106	96	136	29.04
	Missing	9	4	3	1.37
	Not yet specified	409	113	288	69.59
Usage Precautions	Labeled	392	189	310	76.55
	Missing	4	4	3	0.95
	Not yet specified	128	20	114	22.51
Drug Interactions	Labeled	258	111	201	48.97
	Missing	266	102	226	51.03

The adverse reactions of pediatric Chinese patent medicines with labeled warnings were categorized. The primary adverse reactions predominantly involved the Gastrointestinal System, followed by the Skin and its appendages, the Neuropsychiatric System, and systemic damage, as detailed in Table 9.

**TABLE 9:** The system and organs analysis on the adverse reaction of Chinese patent medicines for children.

Category	Pediatric-specific medications	Medications for both children and adults	Medications with dosage reduction for children	Proportion/%
Gastrointestinal System	47	40	39	78.26
Skin and its appendages	18	19	27	39.75
Nervous System	6	13	21	24.84
Systemic Damage	4	14	17	21.74
Cardiovascular System	1	8	6	9.32
Respiratory System	2	6	5	8.07
Administration Site	4	4	4	7.45
Hepatobiliary System	2	5	4	6.83
Urinary System	3	2	2	4.35
Hematologic System	2	2	3	4.35
Others	0	2	2	2.48
Female Reproductive System Damage	0	2	0	1.24
Visual Impairment	0	1	0	0.62

Among pediatric Chinese patent medicines, 27 types (6.33%) have documented clinical trials, including 18 pediatric-specific medications, six medications for both children and adults and three medications with dosage reduction for children. Of these, 24 pharmaceutical products received clinical research approval before 2006, while three were approved in 2006 and thereafter. Seventy-two pediatric Chinese patent medicines (6.19%) specify pharmacological/toxicological effects, comprising 37 pediatric-specific medications, 18 medications for both children and adults and 17 medications with dosage reduction for children. A significant proportion of pediatric Chinese patent medicines lack well-defined safety information, posing potential risks in clinical medication practices.

### 3.8 Inclusion status in the three major directories

**FIGURE 1:**
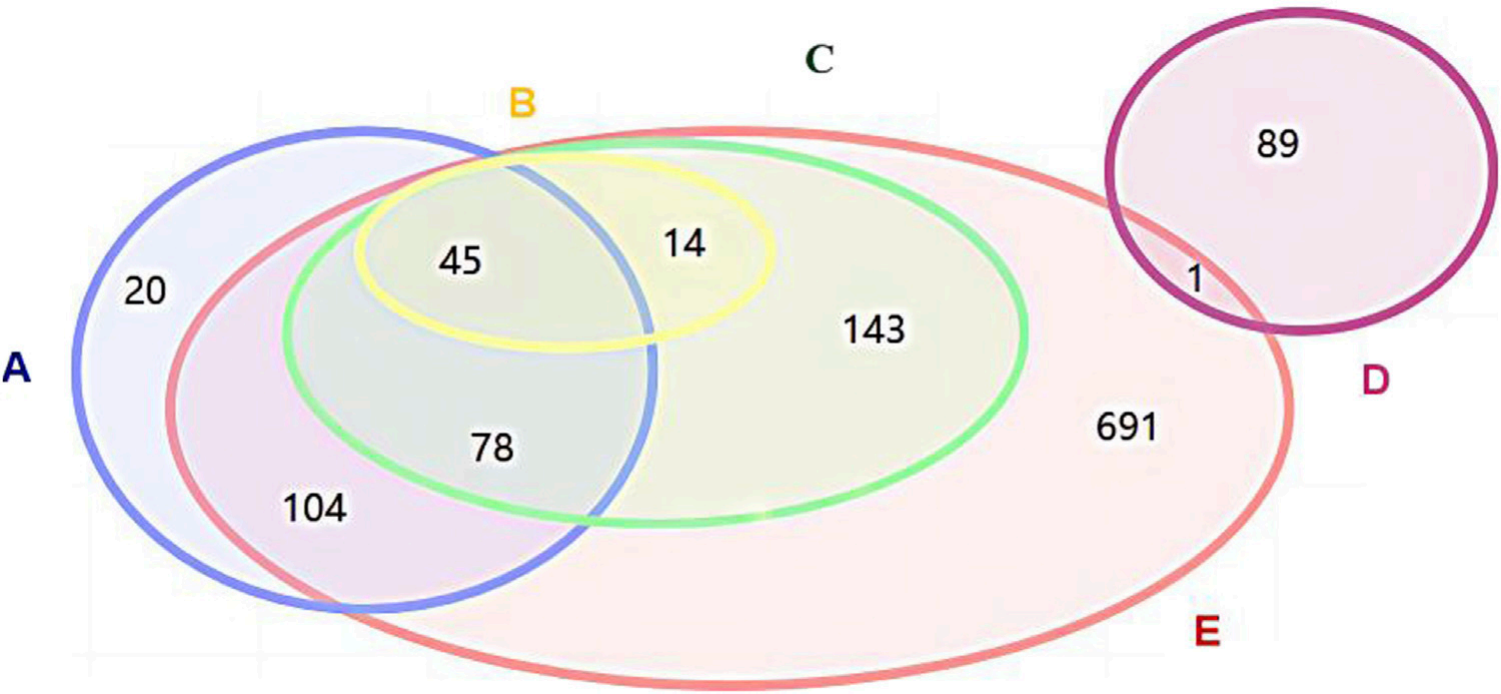
Relationship diagram between Chinese patent medicines for children in the three catalogsand Chinese patent medicines for children in the National Drug Code Database **(A)** Chinese Pharmacopoeia-listed Chinese paten medicines for children; **(B)** Essential Medicines List-included Chinese paten medicines or children; **(C)** National Reimbursement Drug List-covered Chinese paten medicines for children; **(D)** “National Medicine Approval Number B”-Chinese paten medicines for children; **(E)** “National Medicine Approval Number Z”-Chinese paten medicines for children.

Among pediatric Chinese patent medicines with “National Medicine Approval Number Z” approval numbers, 691 products were not included in the three major directories, while none of the “National Medicine Approval Number B” pediatric Chinese patent medicines were listed in these directories; Xiao’er Chiqiao Qingre Granules and Xiao’er Zibei Xuanfei Syrup, both negotiated drugs in the National Reimbursement Drug List (NRDL), were approved for market release in October and November 2023 respectively and thus excluded from this study; Twenty pediatric Chinese patent medicines listed in the Chinese Pharmacopoeia were excluded from the Pediatric Chinese Patent Medicine Database due to either lacking pediatric medication information in their drug package inserts or missing drug approval numbers, as illustrated in Figure 1.

## 4 Actionable recommendations

### 4.1 Conduct post-marketing Re-evaluation

Post-marketing re-evaluation research constitutes a crucial component of pharmaceutical product lifecycle management. Through conducting post-marketing studies on the efficacy, safety, and cost-effectiveness of pediatric Chinese patent medicines (Wang et al., 2024; Shi et al., 2024), we aim to clarify clinical positioning, explore therapeutic value, and enhance safety information for pediatric medications, thereby providing scientific evidence for rational drug use and healthcare decision-making. Furthermore, implementing a clinical comprehensive evaluation of pediatric Chinese patent medicines from multiple dimensions including safety, efficacy, cost-effectiveness, suitability, accessibility, and innovation (Yuan et al., 2023; Zhang et al., 2021; Hu et al., 2024; National Health Commission Development Research Center, 2022) will help define their comprehensive clinical value. This process supports evidence-based development of clinical guidelines and pathways, informs updates to national pharmaceutical catalogs, guides quality improvement initiatives for pediatric Chinese patent medicines, and ultimately contributes to the high-quality development of Chinese patent medicines.

### 4.2 Improve pediatric drug package inserts

Drug package inserts are legal documents containing pharmaceutical information, serving as crucial channels for clinicians and patients to understand drug properties. The standardization of inserts is closely related to rational drug use in clinical practice. To promote the inheritance, innovation, and development of traditional Chinese medicine (TCM) and strengthen the research/registration management of new TCM drugs, the “Special Regulations on the Registration and Administration of Traditional Chinese Medicines” (National Medical Products Administration.2023) Enterprises are advised to enhance lifecycle management of pharmaceutical products, intensify monitoring, evaluation, and analysis of safety risks, and promptly revise/improve package inserts to ensure complete, accurate, and standardized content that guides rational clinical use.

### 4.3 Research and development of pediatric medications new varieties

To promote the inheritance, innovation, and development of traditional Chinese medicine (TCM) and accelerate the approval and marketing of new traditional Chinese medicine drugs, the government has issued a series of policy documents (Center for Drug EvaluationNational Medical Products Administration, 2022; National Medical Products Administration, 2020; Central Committee of the Communist Party of China and State Council, 2019). Guided by the “Three Combinations” principle, efforts are made to clarify the clinical value positioning of clinically validated formulas, hospital preparations, and modified ancient prescriptions for treating or assisting in the management of diseases, syndromes, and symptoms, thereby enabling precision-driven research and development (Wang et al., 2025; Lu et al., 2025; Ma et al., 2022; Luan and Hu, 2022). When conducting clinical trials, appropriate target populations should be selected based on the disease categories, age-appropriate dosage forms should be prioritized for specific age groups, and more standardized clinical trial protocols should be designed to facilitate the translation of clinical research outcomes into practical applications (Yang et al., 2023). By leveraging modern technologies such as network pharmacology to comprehensively understand the complexity of traditional Chinese medicine, we can strengthen the evidence chain, optimize prescriptions, and accelerate the development of new traditional Chinese medicine drugs (Li and Xiao, 2024).

### 4.4 Enhancing the appropriateness of pediatric medications

For products with suboptimal dosage form appropriateness, reference should be made to the Technical Guidelines for Research on Modified New Traditional Chinese Medicine Drugstore reformulate them into more acceptable dosage forms (Center for Drug Evaluation of the National Medical Products Administration, 2024); For products with bitter taste that are poorly tolerated, the Technical Guidelines for Palatability Design and Evaluation of Pediatric Medications should be referenced for dosage form improvements (Jichuan Pharmaceutical Group Co., Ltd., 2019) flavor masking studies (He et al., 2017; Yang et al., 2020), and improvements in preparation processes (Harbin Kanglong Pharmaceutical Co., Ltd., 2020) by improving drug palatability and enhancing compliance, thereby better serving clinical needs; for pharmaceutical products with confirmed clinical efficacy but undefined pediatric dosages, reference should be made to the Technical Guidelines for Model-Informed Drug Development (Center for Drug Evaluation, National Medical Products Administration, 2020b) Technical Guidelines for Extrapolating Adult Drug Data to Pediatric Populations (Center for Drug EvaluationNational Medical Products Administration, 2023) to conduct pediatric Chinese patent medicine dose model construction (Luan et al., 2023; Chen et al., 2021) and clinical trial research (Sun et al., 2021), thereby clarifying medication dosages for children of different ages, providing evidence for modifying pediatric drug specifications, ensuring clinical medication safety for children, and reducing the phenomenon of ‘splitting pills for dosing and guessing doses’ among children.

## 5 Discussion

As of May 2023, China has approved 9,847 types of Chinese patent medicines for marketing, with 1,164 being pediatric Chinese patent medicines, accounting for 11.82% of all approved Chinese patent medicines. National pharmaceutical catalogs currently list only 384 approved pediatric Chinese patent medicines. Data shows that in 2022, pediatric outpatient and emergency visits in medical institutions reached approximately 469.48 million, constituting 7.88% of total visits. The 2018 survey on 2-week morbidity rates revealed: 22.0% for 0–4-year-olds, 13.1% for 5–14-year-olds, and 10.6% for 15–24-year-olds (National Medical Products Administration, 2023). This demonstrates that currently available pediatric Chinese patent medicines in China predominantly consist of dual-use medications for adults and children (64.9%) and medications requiring dosage reduction for children (28.3%), while pediatric-specific medications account for merely 6.8%. However, dual-use medications and dosage-adjusted medications, primarily designed for adult therapeutic needs, may not adequately meet children’s special requirements for dosage forms, specifications, and other pharmaceutical particulars.

As of May 2023, pediatric Chinese patent medicines in China predominantly utilize oral administration, with primary dosage forms including granules, pills, tablets, powder formulations, and capsule preparations. Pediatric-specific medications primarily employ oral administration (95.0%), mainly through granules, pills, powder formulations, and oral liquids. Topical medications account for 3.2% while rectal administration constitutes 0.8%. In December 2020, the Center for Drug Evaluation of the National Medical Products Administration (CDE) The issuance of the ‘Guiding Principles for Pharmaceutical Development of Pediatric Medications (Chemical Drugs) (Trial)' provides examples illustrating the relationship between routes of administration/dosage forms and age for pediatric medications (Center for Drug Evaluation, National Medical Products Administration, 2020a). Analysis based on these guidelines reveals that pediatric Chinese patent medicines predominantly employ dosage forms suitable for older children while exhibiting critical gaps in external-use preparations with transdermal/percutaneous delivery and rectal administration dosage forms appropriate for younger age groups. Notably, these guiding principles specifically address chemical drugs, leaving the field of pediatric Chinese patent medicines without corresponding regulatory frameworks.

Based on pharmaceutical product characteristics information from drug package inserts, the palatability of oral pediatric Chinese patent medicines in China primarily features bitter and sweet flavors, with other undesirable tastes present. Similarly, specialized oral pediatric Chinese patent medicines exhibit predominantly bitter and sweet flavors. Children’s taste perception is more sensitive than adults, with lower bitterness thresholds. Many Chinese medicinal compounds contain bitter components such as alkaloids (e.g., berberine, marine) and flavonoids (baicalin, quercetin), which significantly impede the application of traditional Chinese medicine in pediatrics. While ensuring efficacy and safety, pediatric Chinese patent medicines should comprehensively improve palatability to enhance medication adherence in children, thereby better serving clinical pharmaceutical needs.

Pediatric Chinese patent medicines in China cover multiple therapeutic domains including Pulmonary Disorders, Splenic Disorders, Cardiovascular and Cerebrovascular Diseases, Ear, Nose, and Throat Disorders, Disorders of Qi, Blood, and Body Fluids, Hepatobiliary Disorders, Renal Disorders, and Orthopedic and Traumatic Diseases, with predominant applications in Pulmonary Disorders, Splenic Disorders, and Cardiovascular and Cerebrovascular Diseases. Among healthcare products, pediatric Chinese patent medicines primarily target Splenic Disorders, Cardiovascular and Cerebrovascular Diseases, and Disorders of Qi, Blood, and Body Fluids. Specifically, dedicated pediatric formulations focus predominantly on Splenic Disorders (38.9%) and Pulmonary Disorders (33.0%). This concentration aligns with common pediatric illnesses (Chen et al., 2024; Institute for Drug EconomicsSouth ChinaNational Medical Products Administration, 2016), yet reveals clinical gaps in other therapeutic areas that may require supplementation through Medications for Both Children and Adults and Medications with Dosage Reduction for Children. The uneven or absent distribution of pediatric Chinese patent medicines across therapeutic categories reflects systemic issues of oversupply or insufficiency in these domains, potentially hindering the high-quality development of pediatric traditional Chinese medicine and compromising medication accessibility for pediatric populations.

The completeness and clarity of pediatric medication safety information in the instructions of Chinese patent medicines are closely related to medication safety for children. In this study, pediatric Chinese patent medicines exhibited varying degrees of deficiencies in documenting adverse reactions, contraindications, usage precautions, and drug interactions. The lack of safety information in pediatric Chinese patent medicine instructions reflects insufficient safety research by pharmaceutical enterprises, and inadequate post-marketing safety surveillance, reevaluation, and pharmaceutical product lifecycle management. This indirectly highlights issues in the implementation of the extent {Drug Administration Law of the People’s Republic of China}. 《Work Procedures for Supplementing Pediatric Medication Information in Marketed Drug Package Inserts》《Workflow of the Center for Drug Evaluation on Supplementing Pediatric Medication Information in Marketed Drug Package Inserts》and the implementation level of these regulatory policies. The lack of safety information prevents clinicians and pharmacists from fully understanding crucial information such as adverse reactions, contraindications, usage precautions, and drug interactions when guiding medication use or when caregivers purchase medications for children. This hampers the effective assessment of potential medication risks and poses hidden dangers to pediatric medication. Therefore, to ensure the safety of pediatric medication, it is imperative to improve relevant information in drug package inserts, strengthen regulatory oversight, emphasize adverse reaction monitoring of marketed pharmaceuticals, and enhance the scientificity and rationality of using Chinese patent medicines in children.
